# Patient-Centered Outcomes Research Institute (PCORI) multisite project to develop treatment decision aids for racial/ethnic minorities with lupus nephritis

**DOI:** 10.1186/ar4664

**Published:** 2014-09-18

**Authors:** Jinoos Yazdany, Maria Dall'Era, Jasvinder Singh

**Affiliations:** 1 University of California, San Francisco, CA, USA; 2 University of Alabama at Birmingham, AL, USA

## Background

The Patient-Centered Outcomes Research Institute (PCORI) was established to support research that informs US health reform. The PCORI aims to provide patients and the public with information to facilitate healthcare decision-making, including to 'develop, refine, test, and/or evaluate patient-centered approaches, including decision support tools.' Here we describe the first PCORI project in systemic lupus erythematosus (SLE), in which we aim to develop an individualized patient decision aid for lupus nephritis. The project uses a multiphase approach to develop and test a decision aid, combining comparative effectiveness research, qualitative research with patients, decision-aid prototype development, and a randomized controlled trial.

## Methods

We performed comparative effectiveness research (CER) using state-of-the-art network meta-analyses to assess effectiveness and toxicities of treatments for lupus nephritis. Concurrently, patients with lupus nephritis participated in Nominal Group Technique (NGT) groups. Participants identified facilitators and barriers they faced when considering and taking drugs for lupus nephritis. In the coming year, information from the CER and NGT will be used to create a prototype decision aid, with input from diverse project stakeholders, including design consultants, clinicians, researchers, lupus organizations and patients. We will then perform a randomized controlled trial to assess the effectiveness of the decision aid (vs. usual care) in patients with incident or recurrent lupus nephritis. Primary outcomes include measures of decisional conflict and informed choice. Secondary outcomes include the control preferences scale and interpersonal processes of care, and exploratory outcomes will include medication adherence and persistence, cumulative glucocorticoid exposure, and renal response.

## Results

The CER and NGT have been completed. Fifty-two patients, who were predominantly African American and Hispanic, participated in NGT groups. Patients identified barriers and facilitators to use of immunosuppressive drugs in two 1-hour sessions, each with three to nine patients. Data from CER and NGT are currently being analyzed. A prototype decision aid will be developed based on the results of the CER and NGT, and subsequently piloted in patients before the randomized trial.

## Conclusions

The decision aid resulting from this project will be nonproprietary, free of cost and readily available for use both in the United States and internationally. Use of the tool is expected to facilitate patient-centered care for lupus nephritis by providing information that both scientific evidence and patients themselves indicate is critical before commencing therapy. The project aims to improve decision-making and health outcomes for lupus nephritis, particularly among racial/ethnic minorities.

